# Pivmecillinam with Amoxicillin/Clavulanic acid as step down oral therapy in febrile Urinary Tract Infections caused by ESBL-producing Enterobacterales (PACUTI)

**DOI:** 10.1186/s13063-023-07542-3

**Published:** 2023-09-02

**Authors:** Jonas Tverring, Emeli Månsson, Vigith Andrews, Oskar Ljungquist

**Affiliations:** 1 Department of Clinical Sciences Helsingborg (AKVH), Faculty of Medicine, Lund University, Lund, Sweden; 2 Department of Infectious Diseases, Helsingborg Hospital, Region Skåne, Helsingborg, Sweden; 3 Department of Infectious Diseases and Centre of Clinical Research, Västmanland Hospital, Västerås, Sweden; 4 Department of Clinical Microbiology, Lund University Hospital, Lund, Sweden

**Keywords:** Urinary tract infection, Extended spectrum beta-lactamase, Randomised controlled trial

## Abstract

**Background:**

Oral treatment alternatives for febrile urinary tract infections are limited in the era of increasing antimicrobial resistance. We aim to evaluate if the combination of pivmecillinam and amoxicillin/clavulanic acid is non-inferior to current alternatives for step-down therapy in adult patients with febrile urinary tract infection.

**Methods:**

We plan to perform an investigator-initiated non-inferiority trial. Adult hospitalised patients treated with 1–5 days of intravenous antibiotics for acute febrile urinary tract infection caused by extended spectrum beta-lactamase (ESBL) producing Enterobacterales will be randomised 1:1 to either control (7–10 days of either oral ciprofloxacin 500 mg twice daily or oral trimethoprim–sulfamethoxazole 800 mg/160 mg twice daily or intravenous ertapenem 1 g once daily, depending on sex, drug allergy, glomerular filtration rate and susceptibility testing) or intervention (10 days of pivmecillinam 400 mg three times daily and amoxicillin/clavulanic acid 500/125 mg three times daily). The primary outcome will be clinical cure 10 days (+/− 2 days) after antibiotic treatment completion. Clinical cure is defined as being alive with absence of fever and return to non-infected baseline of urinary tract symptoms without additional antibiotic treatment or re-hospitalisation (for urinary tract infection) based on a centralised allocation-blinded structured telephone interview. We plan to recruit 330 patients to achieve 90% power based on a sample size simulation analysis using a two-group comparison, one-sided alpha of 2.5%, an absolute non-inferiority margin of 10% and expecting 93% clinical cure rate and 10% loss to follow-up. The primary endpoint will be analysed using generalised estimated equations and reported as risk difference for both intention-to-treat and per protocol populations. Patients are planned to be recruited from at least 10 centres in Sweden from 2023 to 2026.

**Discussion:**

If the combination of pivmecillinam and amoxicillin/clavulanic acid is found to be non-inferior to the control drugs there are potential benefits in terms of tolerability, frequency of interactions, outpatient treatment, side effects, nosocomial infections and drive for further antimicrobial resistance compared to existing drugs.

**Trial registration:**

NCT05224401. Registered on February 4, 2022

## Introduction

### Background and rationale {6a}

Antimicrobial resistance (AMR) is increasing. An estimated 4.95 million deaths were associated with AMR worldwide in 2019 and the total cost of health care and loss of productivity exceeds € 1.5 billion every year in the European Union [1, 2]. Extended spectrum beta-lactamase (ESBL) producing *Enterobacterales* (EPE) is of increasing concern, often colonising the gut and intestines, and causing urinary tract infections (UTI) and bloodstream infections (BSI). ESBL-producing bacteria often carry resistance to other antibiotic classes than beta-lactam antibiotics, for instance quinolones and trimethoprim-sulfamethoxazole (TMX), which are the mainstay oral treatment for febrile UTI [3]. This can result in longer treatment durations with intravenous antibiotics, either in hospitals or in outpatient clinics, which leads to greater health care costs, increased risks of nosocomial infections for patients, less patient autonomy and increased risk or acquiring further multidrug-resistant bacteria [4].

Pivmecillinam is the oral prodrug of mecillinam, a beta-lactam discovered in the 1970s with activity against *Enterobacteriaceae* [5]. There are at least three RCTs supporting the use of pivmecillinam for treatment of uncomplicated lower urinary tract infection (UTI) in women [6–8] and one in men [9]. Pivmecillinam has been used for decades in the Nordic countries [3], showing low resistance rates (<5%) [10, 11], high tolerability in general [7] and in pregnant women [12], with little adverse effects on microbiota [13] and a low drive for further resistance [14]. Treating febrile UTI (e.g., pyelonephritis) with pivmecillinam is still controversial due to a lack of evidence from RCTs but is practiced in many institutions in Sweden, Denmark and Norway [15]. A recent prospective observational study from Norway supported the use of pivmecillinam as step-down therapy for pyelonephritis with *E. coli* bacteraemia [16] while a retrospective study of pivmecillinam in EPE-causing pyelonephritis showed high rates of clinical failure [17]. We believe that these observational studies may reflect a true association, i.e. that pivmecillinam may be sufficient for non-EPE but not for EPE. Pivmecillinam has low affinity for many beta-lactamases with preserved activity in vitro [18] but pivmecillinam is sensitive to hyperproduction of beta-lactamases [19] and to the inoculum effect with several-fold increases in MIC [20]. When pivmecillinam is co-administrated with clavulanic acid (inhibiting ESBLs), the low MIC is retained, despite high inoculum, and in vitro efficacy is improved in time-kill experiments [19–23]. There may also be some synergy between pivmecillinam and ampicillin, but the data is divergent [24]. The pivmecillinam and amoxicillin/clavulanic acid (PAC) combination has been evaluated in an observational study as pre- and post-prophylaxis for transrectal ultrasound-guided biopsy of the prostate (TRUBP) and showed lower rates of bacteraemia compared to prophylactic ciprofloxacin and with significantly fewer EPE cultured [25]. Clinical trials for the use of PAC in febrile UTI are lacking however, and a search for the PAC combination in the World Health Organisation’s International Clinical Trials Registry Platform revealed no results.

We hypothesise that PAC is non-inferior to ciprofloxacin, TMX or ertapenem as step-down therapy for acute febrile UTI caused by EPE, based on the assumption that pivmecillinam is an effective treatment for febrile UTI and that the addition of clavulanic acid ensures efficacy in patients with ESBL-producing Enterobacterales.

### Objectives [7]

#### Primary objective

To evaluate if the combination PAC (intervention) is non-inferior to ciprofloxacin, TMX or ertapenem (as one composite control group) as step-down therapy in patients with acute febrile UTI caused by extended spectrum beta-lactamase (ESBL) producing Enterobacterales (EPE).

#### Secondary objectives

1. To compare participants’ perception of treatment tolerability on a 1–10 scale.
2. To compare the incidence of early study drug discontinuation between groups.
3. To compare the incidence of additional antibiotic subscriptions (for UTI) within 28 days between groups.
4. To compare re-admission to hospital (due to UTI-related symptoms) within 28 days between groups.
5. To compare the incidence of drug-related serious adverse events (SAE) within 28 days between groups.
6. To compare the all-cause mortality within 28 days between groups.
7. To compare the recurrence prevalence of EPE (phenotypically same species) in urine cultures 10 +/− 2 days after antibiotic treatment between groups (i.e. microbiological cure).
8. To compare the prevalence of EPE or carbapenemase-producing bacteria in faecal cultures 10 +/− 2 days after antibiotic treatment between groups.

### Trial design {8}

Investigator-initiated, randomised, controlled, investigator-blinded, outcome-assessor-blinded, non-inferiority, multicentre, phase IV trial with parallel group design, 1:1 concealed allocation ratio and a 28-day follow-up period.

## Methods: participants, interventions and outcomes

### Study setting {9}

We will recruit patients from up to 29 infectious disease (ID) clinics in secondary and tertiary, emergency and teaching hospitals in Sweden. Participant recruitment 2023 in the following five Swedish ID clinics: Helsingborg, Malmö, Lund, Kristianstad and Västerås. A full list of currently recruiting centres will be updated on https://classic.clinicaltrials.gov/ct2/show/NCT05224401 and on the trial homepage: https://pacuti.se/.

### Eligibility criteria {10}

Patients will be screened for eligibility by the attending ward physician using the following criteria:

#### Inclusion criteria (all of the following)

1. Age ≥ 18 years
2. Fever (≥ 38.3 °C) or shaking chills at least once at home or in hospital
3. Clinical suspicion of UTI including at least one of the following symptoms:
  a Dysuria, urinary urgency, difficulty urinating, new or worsened urinary incontinence, macroscopic haematuria or increased urinary frequency
  b Low abdominal pain or flank pain with percussion or palpation tenderness over kidneys and/or bladder.
4. Urine (≥ 10^3^ CFU/mL) and/or blood culture positive for EPE^1^ with susceptibility to pivmecillinam^2^.
5. In-patient who has received 1–5 days of EPE-active^3^ intravenous antibiotics
6. Discontinuing parenteral treatment and starting treatment with oral antibiotics is considered safe according to the treating physician.

#### Exclusion criteria (any of the following)

1. Known or suspected pregnancy.
2. Known or suspected life-threatening allergy towards beta-lactam antibiotics.
3. Clinical isolate of EPE is resistant to ciprofloxacin, TMX and ertapenem.
4. Severe renal insufficiency with estimated glomerular filtration rate (eGFR) <10 mL/min or requiring any form of dialysis.
5. Severe decompensated liver failure (i.e. child Pugh class B or C).
6. Genetic metabolic diseases associated with severe carnitine deficiency.
7. Megaloblastic haematopoiesis.
8. Co-treatment with valproate or valproic acid (due to interaction with pivmecillinam and ertapenem, respectively)
9. Other reason to which patient is unfit to be included in the study according to treating physician, e.g. cognitive impairment preventing informed consent and follow-up, inability to speak and/or read Swedish, missing national personal identification number or missing telephone number preventing follow-up or planned duration of antibiotics >10 days due to complicating factors.

### Who will take informed consent? {26a}

A limited number of infectious disease physicians at each site will be responsible for obtaining informed consent from trial participants prior to treatment allocation. These physicians will receive specific training and instructions on how to follow protocol and how to collect and record data in the trial. The physicians will provide oral and written information about the study, any risks or benefits involved with participation and inform potential participants that termination from the study can be done at any time without explanation in accordance with the ethical approval. Written information will be provided after all eligibility criteria is fulfilled on days 1–5 from the initiation of EPE-active intravenous antibiotic. Recruiting physicians are asked to provide at least a few hours for each participant to contemplate their participation.

### Additional consent provisions for collection and use of participant data and biological specimens {26b}

No additional participant data or biological specimens will be collected other than what is specified in this protocol. The data collected for this study may be used in ancillary studies after approval of an amendment to the Swedish ethical review authority.

## Interventions

### Explanation for the choice of comparators {6b}

#### Drug choice

Fluoroquinolones and trimethoprim–sulfamethoxazole are considered international standard oral treatment for febrile UTI [3] and have been used since more than 20 years [28]. We use oral ciprofloxacin (500 mg twice daily) in our study based on local tradition, but oral levofloxacin (750 mg once daily) would have been an equally good choice [29]. Oral fluoroquinolones can be considered superior to oral trimethoprim–sulfamethoxazole (800 mg/160 mg twice daily) as an empirical therapy based on higher resistance rates for trimethoprim–sulfamethoxazole [30] but they are considered equally good choices when resistance patterns are known, which will be the case in our study [3]. For EPE strains with resistance to both ciprofloxacin and TMX (probably >30% of samples), the study protocol recommends intravenous ertapenem (1 g once daily). Ertapenem has been evaluated in at least 4 randomised controlled trials (RCTs) and proved equally effective and tolerable for the treatment of complicated UTI as a traditional treatment with a third-generation cephalosporin [31–33]. Ertapenem is widely used as once daily intravenous outpatient treatment of EPE-caused febrile UTIs with retrospective data supporting good clinical outcomes [34, 35] that are comparable to TMX [36].

#### Treatment duration

A 7-day course of ciprofloxacin has been shown to be non-inferior to a 14-day course in women in an RCT [37]. There is retrospective data indicating that a 7-day course may also be adequate for trimethoprim–sulfamethoxazole [38] but data from randomised trials are lacking and in practice a 10-day course in often used [39]. In men, a 2-week course of ciprofloxacin was non-inferior to a 4-week course [40]. In a more recent trial, a 7-day course of ciprofloxacin was inferior to a 14-day course of ciprofloxacin in short-time follow-up (10–18 days) but not in long-term follow-up (70–84 days post treatment) [41]. In a randomised trial of afebrile and haemodynamically stable patients with gram-negative bacteraemia where 47% were men and 68% had UTI, 7 days of antibiotics was non-inferior to 14 days [42]. In clinical practice [28] and for the purpose of our study, we consider a total treatment duration of 10 days to be reasonable for all drugs except ciprofloxacin in women where 7 days have been proved to be equally effective [37].

### Intervention description {11a}

#### Control

After 1–5 days of EPE-active intravenous antibiotics switchover to treatment^4^ totalling 10 days of either oral ciprofloxacin (500 mg twice daily^5^) or oral TMX (800 mg/160mg twice daily^6^) or intravenous ertapenem^5^ (1 g once daily^5^) for men and 7 days of ciprofloxacin (500 mg twice daily) or^6^ 10 days oral TMX (800 mg/160 mg twice daily) or^6^ intravenous ertapenem (1 g once daily) for women (Table 1 and 2).

**Table 1:** Protocol-suggested drug in control group according to susceptibility

Susceptibility	Ciprofloxacin S	Ciprofloxacin R
TMX S	TMX^a^ (~25%)	TMX^a^ (~10%)
TMX R	Ciprofloxacin^b^ (~25%)	Ertapenem (~40%)

*S* Susceptible, *R* Resistant, according to EUCAST breakpoints [26]. Percentages refer to the *expected* (unpublished) allocation of patients between the different groups in the study, i.e. approximately total TMX 35%, Ciprofloxacin 25% and Ertapenem 40%

^a^Participants with eGFR < 20 ml/min should receive ciprofloxacin or ertapenem instead, depending on susceptibility testing and any drug allergy and/or co-treatments

^b^Participants with suspected or known aortic aneurysm, myasthenia gravis or long QT-syndrome should be treated with TMX or ertapenem depending on susceptibility testing and any drug allergy and/or co-treatments

**Table 2:** Overview of drug interactions

**Study drug**	**Co-medication/s**	**Expected effect**
Amoxicillin/clavulanic acid	Acenocoumarol, Warfarin	Increased INR. Monitor regularly
Amoxicillin/clavulanic acid	Methotrexate	Increased concentration of co-medication
Amoxicillin/clavulanic acid	Probenecid	Increased concentration of study drug
Amoxicillin/clavulanic acid	Mycophenolic acid	Decreased concentration of co-medication
Pivmecillinam	Probenecid	Increased concentration of study drug
Pivmecillinam	Methotrexate	Increased concentration of co-medication
Pivmecillinam	Valproate/valproic acid	Both drugs decrease carnitine levels which could increase risk for hyperammonaemia
Ciprofloxacin	Class IA and III antiarrhythmics, tricyclic antidepressants, macrolides, antipsychotics	Both study drug and co-medication lead to QT-elongation
Ciprofloxacin	Probenecid, Omeprazol	Increased concentration of study drug
Ciprofloxacin	Tizanidine, Methotrexate, Duloxetine, Ropinirole, Clozapine, Sildenafil, Agomelatine, Zolpidem, Theophylline, Phenytoin, Glibenclamide	Increased concentration of co-medication. Consider therapeutic drug monitoring.
Ciprofloxacin	Cyclosporine	Increase in serum creatinine
Ciprofloxacin	Vitamin K-antagonists	Increase in anticoagulant effect. Monitor INR.
Trimetoprim–sulfamethoxazole	Dofetilid, Amantadine, Digoxin, Memantine, Metformin, Methotrexate, Lamivudine, Paclitaxel, Amiodarone, Dapsone, Repaglinide, Rosiglitazone, Pioglitazone, Coumarins, Phenytoin. Sulfonylurea derivatives	Increased serum concentration of co-medication. Consider therapeutic drug monitoring.
Trimetoprim–sulfamethoxazole	Clozapine	Risk of agranulocytosis
Trimetoprim–sulfamethoxazole	Ciclosporin, Tacrolimus	Risk of decreased kidney function in kidney transplanted patients
Trimetoprim–sulfamethoxazole	Zidovudine, Azathioprine, Mercaptopurine	Risk of haematological dysfunction
Trimetoprim–sulfamethoxazole	ACE-inhibitors, Angiotensin receptor blockers, Potassium saving diuretics, Prednisolone	Risk of hyperkalaemia
Trimetoprim–sulfamethoxazole	Thiazides	Risk of thrombocytopenia
Trimetoprim–sulfamethoxazole	Tricyclic antidepressants	Decreased concentration of co-medication
Trimetoprim–sulfamethoxazole	Pyrimethamine	Risk of anaemia
Ertapenem	Valproate/Valproic acid	Decreased concentration of co-medication

#### Intervention

After 1–5 days of EPE-active intravenous antibiotics switch-over to treatment totalling 10 days of oral pivmecillinam (400 mg three times daily^5^) and oral amoxicillin/clavulanic acid (500/125 mg three times daily^5^) for both men and women.

### Criteria for discontinuing or modifying allocated interventions {11b}

There are no mandated criteria for discontinuing or modifying allocated interventions, but the patient and the treating physician may choose to do so at any time, upon which they will be asked to report timing and reason for non-adherence.

### Strategies to improve adherence to interventions {11c}

Intervention adherence while in hospital will be monitored by the treating physician and any deviance will be required to be registered in the electronic case report form (eCRF). After patients are dismissed, the allocation-blinded outcome-assessor (the research nurse) will ask the participant whether he or she has taken all the medicines as prescribed, if not why, and how the participant perceived the treatment tolerability, and if any he or she have received any further antibiotic prescriptions for UTI since recruitment to the trial. Even though this pragmatic monitoring cannot guarantee adherence with certainty, it is similar to the how the medications would be used in the clinic. The recruiting physician will further validate that the participants have collected their study drug prescription, and that they have not received any further prescriptions, during the retrospective medical journal review.

### Relevant concomitant care permitted or prohibited during the trial {11d}

We make no restrictions on concomitant care or interventions during the trial.

### Provisions for post-trial care {30}

We have no plan for ancillary or post-trial care or compensation for the participants. This is a pragmatic trial comparing widely used medications.

### Outcomes [12]

#### Primary outcome

Clinical cure 10 days (+/− 2 days, i.e. weekends) after antibiotic treatment completion defined as being alive with absence of fever (≥ 38.3 °C) and resolution of, or return to non-infected baseline of, urinary tract symptoms (as defined in inclusion criteria) without hospital readmission (for UTI symptoms) or additional antibiotic treatment (for UTI symptoms) based on a structured telephone interview performed by a centralised allocation-blinded study nurse.

#### Secondary outcomes

1. Participants’ perception of treatment tolerability on a 1–10 scale.
2. Early study drug discontinuation.
3. Additional antibiotic subscriptions (for UTI) within 28 days from randomisation.
4. Proportion of patients re-admitted to hospital (due to UTI symptoms) within 28 days from randomisation.
5. Drug-related serious adverse events within 28 days from randomisation.
6. All-cause mortality within 28 days from randomisation.
7. Growth of EPE (phenotypically same species) in urine culture (10^3^ CFU/mL) 10 +/− 2 days after antibiotic treatment.
8. Growth of EPE or carbapenemase-producing bacteria in faecal cultures 10 +/− 2 days after antibiotic treatment.

### Participant timeline [13]

The trial will end after 28 days from study recruitment (see Table 3 below). Any additional care for the trial participants once their participation in the trial has ended, where it differs from what is normally expected according to the medical condition of the clinical trial participant, will be managed according to routine care within Swedish healthcare. All documents will be stored for a minimum of 25 years after the trial has ended.

**Table 3:**
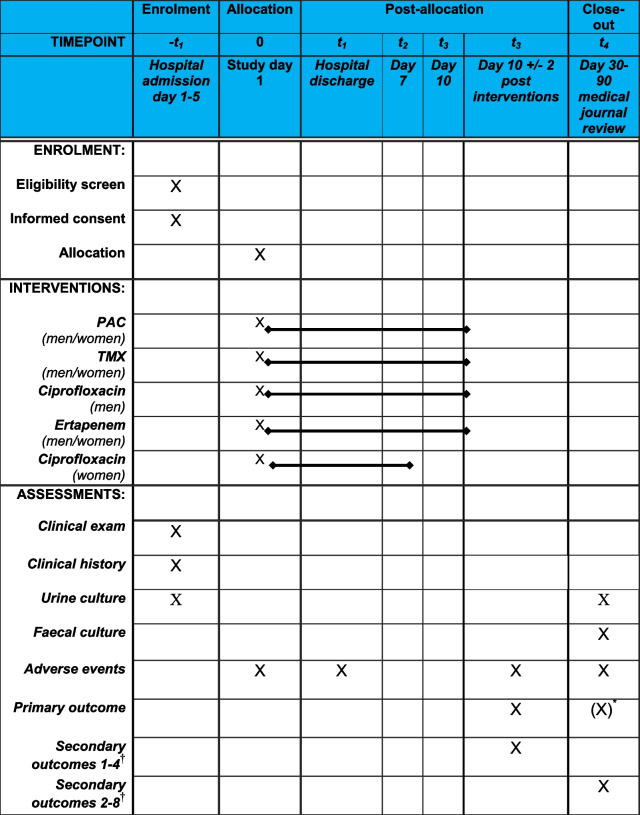
Participant timeline

*PAC pivmecillinam and amoxicillin/clavulanic acid, TMX trimethoprim-sulfamethoxazole*

^*^This refers to a sensitivity analysis for the primary outcome using retrospective medical journal review by the treating physician

^†^If information from the telephone interview assessor and the medical chart review assessor are divergent, we will always choose the conservative conclusion and stipulate failure (i.e. 1/0 1)

## Sample size {14}

We expect a clinical cure rate of 93%, based on previous trials on antibiotic efficacy in febrile UTI exploring similar primary outcomes [37, 41]. We have chosen a classic absolute non-inferiority margin of 10% in accordance with the U.S. Food and Drug Administrations (FDAs) recommendation on developing new drugs for complicated UTIs [43]. We consider this to be suitable considering the low severity of all aspects of the composite primary endpoint except for 28-day mortality which we will evaluate specifically as a secondary endpoint and in the interim analysis. When using a one-sided alpha of 2.5% and performing a statistical simulation of 300 enrolled patients in 30 clinics with an intraclass correlation coefficient of 0.1 and generalised estimated equations (GEE) comparison between two groups with a random intercept for site as the primary analysis we reach 90% power. We plan on recruiting 330 patients to allow for 10% loss to follow-up. The study is not powered to infer non-inferiority between the intervention and any single antibiotic in the control group, but only towards the control group as a whole. The secondary objectives should be considered exploratory and are not based on sample size calculations.

### Recruitment {15}

We aim to establish a surveillance or alert system at each microbiological department of participating centres so that all individuals with an EPE-positive urine and/or blood cultures goes through an initial screening for eligibility (i.e. in-patient and susceptibility to mecillinam and susceptibility to at least one of ciprofloxacin, TMX or ertapenem). If preliminarily eligible, the microbiologist will inform the locally recruitment responsible infectious disease physician who will then screen the remaining inclusion and exclusion criteria. Participants can also be recruited directly by an infectious disease physician at each site if they find a patient that is eligible and was not detected by the surveillance system.

We estimate that recruitment of 330 participants will take 3 to 4 years based on the following unpublished data. There were 190 EPE-positive cultures from 130 adults in Region Västmanland (280,000 inhabitants), Sweden, in year 2020. This corresponds to 46 persons per 100,000 inhabitants per year. The catchment area for the eight sites that have so far agreed to recruit patients adds up to just above 2 million persons. If we assume that one in five persons with a positive EPE-culture is eligible for the study and one in two eligible persons can be recruited, it will take 3.6 years to complete recruitment for the trial.

## Assignment of interventions: allocation

### Sequence generation {16a}

We will use a computer-generated blocked random number sequence with stratification for sex and site. We have chosen to stratify by sex because of diverging results in some previous trials for males [37] and females [41]. We are not certain that this represents a true relationship, but it is something that we would like to evaluate as a fixed effect in the primary analysis and hence balance in the allocation sequence [44]. The sequence and block size will be unavailable to those who enrol participants and to the trial investigators.

### Concealment mechanism {16b}

The allocation sequence will be implemented into, and concealed within, REDCap’s internet-based randomisation application [45].

### Implementation {16c}

An independent statistician from Clinical Studies Sweden—Forum South [46] will generate the allocation sequence. A limited number of attending infectious disease physicians at each site will be able to enrol participants and assign interventions in REDCap’s randomisation application where the allocation sequence is concealed.

## Assignment of interventions: blinding

### Who will be blinded {17a}

Trial participants and in-patient care providers will not be blinded to interventions in this pragmatic trial. Neither will the treating physician who assesses secondary endpoints 2-8. However, the central outcome assessor of the primary outcome will be blinded to interventions, as will the trial investigators, data analysts and author group. When data is extracted from the REDCap database, the allocation will be coded as “1” and “2” and the Code Key will only be revealed after statistical evaluation of the primary and secondary endpoints.

### Procedure for unblinding if needed {17b}

Because care providers and patients are not blinded, there is no reason to unblind for safety of care. The Data Monitoring Committee (DMC) will be able to unblind the database if there are clearly more reports of possibly or probably study drug-related serious adverse events than expected.

## Data collection and management

### Plans for assessment and collection of outcomes {18a}

Baseline data and allocation will be entered into the REDCap database using a short and study-specific electronic case report form (eCRF) directly when assessing eligibility and assigning allocation by the physician who recruited the participant. The trial investigators will instruct a limited number of physicians at participating centre how to access and fill out the eCRF and provide a step-by-step guide and contact information for technical assistance. The primary outcome and the secondary outcomes 2-4 will be assessed using a semi-structured interview with a trial-specific guideline and entered into a separate part of the REDCap eCRF. The first 10 participants’ interview responses will be checked for conformity between at least two study nurses. The remaining secondary outcomes will be assessed retrospectively through medical chart review and from microbiological results by the recruiting physician at 30–60 days after the patient was allocated and then entered into a final separate part of the eCRF. The retrospective chart review will also serve as a sensitivity analysis to the telephone interview for the primary outcome and the secondary outcomes of early drug discontinuation and further antibiotic prescription. A copy of the REDCap eCRF (English) and semi-structured interview guideline (Swedish) can be sent on request.

### Plans to promote participant retention and complete follow-up {18b}

Since the follow-up is via telephone and done centrally, we expect that retention will be high. If the central outcome assessor cannot reach the participant, they will contact the recruiting physician who will check if the participant is deceased through the medical chart. Participants who are not reported dead and still cannot to be reached for the telephone interview follow-up will not qualify for analysis of the primary outcome or bacterial culture-related outcomes but can be assessed retrospectively from medical chart review regarding 28-day mortality, which is important for the evaluation of harm. We suspect that patients who deviate from the prescribed intervention will often be categorised as a negative outcome in the primary outcome assessment (e.g. new antibiotic prescribed), while some will simply be regarded as protocol violations. However, if a participant chooses to discontinue the trial, any collected data will be discarded, and the participant will only appear as a figure in the study flow chart.

### Data management {19}

All data entry will be done electronically, except for code keys and informed consent forms. The eCRF in the REDCap database uses data range checks. The primary outcome and secondary outcomes 2-4 will be double-checked retrospectively against the medical journal by the treating physician 30–60 days after randomisation. Individual patient data will be handled as ordinary chart records and will be kept according to the European General Data Protection Regulation (GDPR). All original records (i.e. code keys and consent forms) will be retained in a locked cabinet at each participating centres infectious disease department for 25 years to allow inspection by relevant authorities. The coded trial database will be maintained for 25 years if requested for revision. Standardised data management procedures can be provided on request from Clinical Trials Sweden—Forum South.

### Confidentiality {27}

At the time of randomisation, participants will be assigned a trial ID number which will be used on all the personal information containing documents (i.e., in consent forms and eCRF). The code key will be kept in a locked cabinet at each participating centre which can only be accessed by the limited number of physicians who can recruit patients. The coded information will be kept in an electronic database which requires two-step verification for login. When data from the study is published in a peer-reviewed medical journal, personal data will not be identifiable.

### Plans for collection, laboratory evaluation and storage of biological specimens for genetic or molecular analysis in this trial/future use {33}

Post-treatment urine and rectal specimens will be financed by the trial but sent to each participating centre’s microbiological laboratory and will be evaluated as per clinical routine and susceptibility testing according to EUCAST guidelines. Only culture positive findings of EPE are noted, and samples will be discarded after 2 weeks from collection without further analysis. Isolates from original urine and blood samples collected from participants at the coordinating microbiology centre (Lund) will be saved in the microbiology department’s freezer storage for preliminary future study of PAC MIC.

## Statistical methods

### Statistical methods for primary and secondary outcomes {20a}

The primary outcome will be analysed between two groups using generalised estimated equations (GEE) using an identity link and binomial family [47], with site as a random effect and sex as a fixed effect. The result will be presented as risk difference with 95% confidence interval. We will state non-inferiority if the lower bound confidence interval does not stretch below −10% risk difference. The first secondary outcome on patients’ perception (ordinal 1–10 scale) will be analysed using a non-parametric Mann Whitney *U* test. The remaining secondary outcomes (all binary) will also be analysed using GEE and reported as a risk difference with 95% confidence interval. Risk differences will be presented in a forest plot. If the telephone interview and the retrospective review report different parameters for the primary outcome (e.g. the patient does not report additional antibiotic prescriptions for UTI, but such prescriptions are identified in the medical chart review), we will always choose the conservative option and consider this a failure.

### Interim analyses {21b}

An interim analysis will be performed once half of the patients have been included (*n* = 165). Blinded data will be delivered by an independent statistician to the Data Monitoring Committee (DMC) who will then provide an allocation-blinded recommendation to the trial investigators whether to continue, hold or terminate the trial prematurely. The recommendation is based on safety and futility. Safety is based on three parameters, the number of possibly related serious adverse events, the number of early study drug discontinuations and the number of deaths within 28 days. It is difficult to provide fixed stopping criteria for safety since these events are expected to be very few, and the DMC have to assess frequency, severity and possible relationship to the study drug and give their best advice accordingly. Futility is defined as less than 5% probability of finding non-inferiority in the final analysis based on conditional power simulations as suggested by Bratton et al. [48]. The trial investigators will make the final decision whether to terminate the trial and will have to provide a written account to explain if they do not follow the recommendation from the DMC.

### Methods for additional analyses (e.g. subgroup analyses) {20b}

We will perform five sensitivity analysis to the primary outcome analysis. One will add a fixed effect (“adjust”) for the number of days of intravenous antibiotics (as a continuous variable) prior to allocation in addition to the baseline GEE model with a fixed effect for sex and a random effect for site. Secondly, we will perform an unadjusted analysis without fixed or random effects. Third, we will also analyse and report the primary outcome based on retrospective chart review versus results from the primary telephone interview. Fourth, we will also perform and report the primary outcome analysis results based on subgroups for the three different control drug allocations (i.e. ciprofloxacin, TMX and ertapenem). Lastly, we will analyse and report results for each sex separately. The last two analyses mentioned are underpowered and cannot provide base for stating individual drug non-inferiority but could provide information of interest for future trial designs.

### Methods in analysis to handle protocol non-adherence and any statistical methods to handle missing data {20c}

We will analyse the population both as intention-to-treat (ITT, i.e. as randomised), modified intention-to-treat (mITT, i.e. patient who took at least one dose of allocated study medication) and per protocol (PP, i.e. no major protocol deviations and missed a maximum of two doses of allocated study drug). We will only state non-inferiority if all three types of analyses provide concordant results [49]. If results are discordant, it will require a close examination of the data and could lead to either a statement of conditional non-inferiority or final uncertainty by the steering committee (SC).

All variables will be screened for frequency and type of missingness (i.e. missing completely at random, missing at random or missing not at random). Multiple imputation with chained equations will be used if missingness is above 5% in any variable included in the primary or secondary analysis. In the case of missingness, data with multiple imputation will be regarded as the primary analysis and complete case analysis will be performed as a sensitivity analysis.

### Plans to give access to the full protocol, participant level-data and statistical code {31c}

This protocol is planned for publication in an open access journal. We plan to publish the statistical code for the primary and secondary outcome analyses as a supplement to the main manuscript publication. Participant-level data can be accessed from the corresponding author on reasonable request.

## Oversight and monitoring

### Composition of the coordinating centre and trial steering committee {5d}

The trial steering committee (SC) consists of the principal investigator (OL) and the co-principal investigator (JT) who will meet in-person or via video link at least on a monthly basis. They are responsible for overseeing the trial and disseminating any changes. At each site, a small team of physician will be instructed and given access to the protocol, study flow chart and the eCRF so that they can recruit patients. They will receive at least one introductory trial-specific education by a member of the protocol author group on site or via video link. The SC will also be available via e-mail, work phone number or video link for questions from the locally responsible trial physician at any of the sites, on a day-to-day basis if needed. On-site responsible physician will receive study updates at least every 6 months. The primary outcome assessor will have access to the SC on a weekly basis.

### Composition of the data safety and monitoring committee, its role and reporting structure {21a}

The Data Monitoring Committee (DMC) will consist of three physician scientists and one biostatistician. Professor Niklas Nielsen will lead the DMC. He has extensive experience with running RCTs and is a professor and consultant in intensive care. Jonas Öberg, specialist in infectious disease, and Erik Senneby, PhD and specialist in clinical microbiology, will also be members of the DMC. The investigators are jointly responsible with the DMC for safeguarding the interests of participating patients and for the conduct of the trial. The DMC will be advisory to the trial investigators regarding the safety and efficacy of the trial. The DMC will be responsible to make a recommendation from blinded data at the interim analysis regarding safety and futility (see above). They will also be informed about serious adverse events (SAE) reported by the trial investigators on a half year basis but can request additional monitoring for SAE at any time. The DMC will meet live at least once for the interim analysis and are recommended to have online meetings at least every half year when SAE reports are delivered. The DMC can request unblinding from the SC if they believe that it is needed to make a valid recommendation.

### Adverse event reporting and harms [22]

Because the trial evaluates well-known and well-used drugs, we have an agreement with the Swedish Medical Products Agency to primarily focus on severe adverse events (SAE, see definition in Table 4 below). The recruiting trial physician will have the primary responsibility to identify and report any SAE from allocation and until day 28. If the telephone interviewer suspects an SAE, they are required to contact the recruiting physician by telephone the same day. If the recruiting physician identifies an SAE, then he or she is required to email the investigators as soon as possible or at latest within 24 h. If a connection between the SAE and the study drug cannot be ruled out, the investigators are responsible to report to the sponsor within 24 h. The sponsor is responsible for ensuring that a serious unwanted and unexpected incident or reaction, which is suspected to be related to the study drug (SUSAR = suspected unexpected serious adverse reaction) is reported to the Medical Products Agency (registrator@mpa.se) on a CIOMS form. SUSARs that are life-threatening or fatal are reported within 7 days to the Medical Products Agency and the Ethical Review Board, and other SUSARs are reported within 15 days. In this study, the Medical Products Agency registers SUSAR in the European trial module EudraVigilance. As long as the trial is ongoing, a safety report will be compiled once a year and reported via EudraCT to the Medical Products Agency.

**Table 4:** Definition of adverse events

Intensity of incident	• Mild does not affect the patient's normal functions • Moderate affects the patient's normal functions to some extent • Severe affects the patient's normal functions to a great extent
Assessment of the connection between incident and study drugs	• Not (unlikely) related to the study drug: there is very little or no possibility that the study drug has caused the unwanted event. • Possibly related to the study drug: the association between the adverse event and the study drug is unknown but there is no other clear cause for the symptoms. • Probably related to the study drug: a reasonable temporal relationship exists between the adverse event and the study drug. Based on the investigator's clinical experience, the association is considered probable.
Serious adverse event	Definition: Any incident or reaction that results in • deaths • is life threatening • requires hospital care or extends an already started care session in hospital • causes a permanent or significant disability or disability • constitutes a congenital malformation or congenital defect.

Recruiting physicians are also encouraged to report non-serious incidents in a specific AE form in the eCRF at any time during hospitalisation or during the retrospective chart review. An incident (adverse event) is defined as any adverse medical event in a patient who has received a study drug. Non-serious incidents are further divided into mild, moderate or severe (as defined below). Relationship between incident and study drug are defined unlikely, possible or probable, as defined below. Incidents that are judged to be related to the study drug will be followed up to enable an assessment of reversibility.

### Frequency and plans for auditing trial conduct [23]

Clinical Trials Sweden—Forum South will monitor the trial with a risk-based approach in accordance with ICH GCP E6(R2). The monitoring purpose is to safeguard the study participants rights and well-being, the validity and completeness of study data collection and to ensure that the study conduct is in accordance with the protocol and ethical and regulatory demands. Monitors will have a live or web-based start-up meeting with each participating site before the study is initiated and conduct two monitor visits during the 3–4 years of patient recruitment for the trial. The first visit will be after 2–5 participants have been recruited and the second visit after 10 additional participants have been recruited, or after 1 year if less than 10 participants have been recruited. The primary monitoring focus will be on informed consent, adverse events reporting and consistency of data entry into the eCRF. The monitor will remind recruiting physician to do the retrospective review. If the monitor discovers major deviations from the protocol, further monitoring visits will be planned. Clinical Trials Sweden—Forum South is a collaboration between Sweden's six healthcare regions (including the sponsor) and is supported and funded by the Swedish Research Council and is independent from the trial investigators. The study will be performed in compliance with the study protocol, the Declaration of Helsinki, ICH-GCP (Good Clinical Practice) guidelines and current national and international regulations governing this clinical trial. In case of an inspection or an audit, the investigator will give direct access to source data in the trial to representatives of the regulatory authority (in case of an inspection) and to the representatives of the sponsor (in case of an audit).

### Plans for communicating important protocol amendments to relevant parties (e.g. trial participants, ethical committees) {25}

The steering committee are responsible for disseminating any important protocol modifications to the ethical board, the Medical Products Agency, the monitoring agent, locally responsible physicians and trial participants in accordance with which party is affected. Extensive changes or safety concerns will be communicated directly by phone and moderate changes by e-mail within 1 week. If there are no changes and the trial runs as planned, we will update site responsible physicians in the least every 6 months. All trial updates will also be posted on the trial-specific homepage www.pacuti.se .

### Dissemination plans {31a}

We plan to publish the trial results in a peer-reviewed medical journal.

## Discussion

### Methodology

Choosing a non-inferiority design always constitutes a compromise in scientific validity compared to a placebo-controlled superiority trial. However, giving patients with serious infections placebo is not ethical, and because EPE-causing febrile UTI is (yet) uncommon in Sweden, we considered a non-inferiority trial to be the best compromise. This enables a high possibility for completing the trial with our available resources and within a reasonable timeframe. The implementation of a surveillance system for EPE in urine and blood cultures in Swedish microbiology departments should be an effective method to ensure consistent recruitment.

Preferably, the treating and recruiting physician and other healthcare personnel would be blinded to treatment allocation. With our design, using three oral and one intravenous antimicrobial, with one to three doses daily, it was overwhelming to us, both in complexity and cost, to design a completely blinded intervention. This non-blinding may affect how patients are treated and how the protocol is adhered. Part of this bias may be managed by analysing the primary outcome both as ITT, mITT and PP. We have put efforts into keeping the primary outcome assessor blinded and stringent in their assessment through the use of a few trained centralised assessors using a study-specific structured interview.

We have chosen to evaluate microbiological cure as a secondary outcome and not include it in the composite clinical cure outcome in contrast to a recently published comparable trial [50]. This choice was not an obvious one. Including microbiological eradication in the composite outcome could increase the objective aspect of a chiefly subjective outcome. We concluded however that mixing clinical cure and microbiological cure could introduce a risk of inaccuracy towards what we are really looking to evaluate, i.e. whether the patient is alive and well. Microbiological eradication is intuitively desirable but in lack of clinical symptoms of UTI empirical data from studies of asymptomatic bacteriuria (ASB) paint a different picture. These tell us that short-term microbiological resolution is often not sustained [51] and that ASB is not necessarily a predictor of symptomatic urinary tract infection (UTI) [52] and may even be protective [53]. Furthermore, follow-up urine cultures are outside of routine management for UTIs in Sweden and we would like the primary outcome to be pragmatic and mirror clinical practice. Any substantial discrepancy between clinical cure and microbiological cure between intervention and control will warrant further investigation, however.

### Benefit versus harm

We consider there to be a low risk of harm from participating in this trial since all drug-specific medications are well-known and widely used. The major risk from a patient perspective is if the intervention is inferior to control. We try to minimise this potential for harm through an interim analysis once half of the intended population has been randomised.

If this trial can show non-inferiority, the potential benefit is the addition of another safe, well-tolerated treatment alternative in the era of antimicrobial resistance. This could, in the best of worlds, improve patient autonomy, lower health-care costs and decrease the drive for further resistance.

## Trial status

Protocol version 1.0 (1 December 2020): first protocol draft

Protocol version 2.0 (21 January 2021): lightly revised protocol sent for ethical and Swedish Medicine Agency approval

Protocol version 3.0 (10 Sept 2021): major protocol revision to fit Trials journal guidelines and sample size update to 330 participants

Protocol version 4.0 (5 May 2022): references update and changed responsibilities of trial statistician

Protocol version 5.0 (30 Sept 2022): major changes in primary outcome evaluation from live visit to centralised telephone interview and addition of third alternative (ertapenem) in control group

Protocol version 5.1 (7 Oct 2022): updates on funding and microbiological sampling

Protocol version 5.2 (27 Oct 2022): updates on DMC responsibilities

Protocol version 5.3 (15 Nov 2022): update on endpoint definition and outcome assessment if discordance

Protocol version 6.1-6.2 (15 Dec 2022): update on adverse events and interactions by request from the Swedish Medical Products Agency

Protocol version 6.3 (31 Jan 2023): updated background with PAC rationale

Protocol version 6.4 (27 Feb 2023): final revision after comments by co-authors

Protocol version 6.5 (13 July 2023, current): minor revision at *Trials* journal regarding SPIRIT item 32, reference checking and an elaboration regarding the choice not to include microbiological cure in the primary composite outcome and a clarification that the statistical analysis is between two groups even though the control group comprises three different drugs.

The first study participant was recruited on 19 April 2023 and recruitment is expected to be completed in year 2026.

## Data Availability

The final trial datasets will be available in its entirety to the trial investigators without any contractual limitations.

## Abbreviations

AE: Adverse events
AMR: Antimicrobial resistance
BSI: Bloodstream infections
DMC: Data Monitoring Committee
eCRF: Electronic case report form
eGFR: Estimated glomerular filtration rate
EPE: ESBL-producing Enterobacterales
ESBL: Extended spectrum beta-lactamase
EUCAST: European Committee of Antimicrobial Susceptibility Testing
FDA: U.S. Federal Drug Administration
GDPR: European General Data Protection Regulation
GEE: Generalised estimated equations
ID: Infectious disease
ITT: Intention-to-treat
mITT: Modified intention-to-treat
OPAT: Outpatient parenteral antimicrobial therapy
PAC: Pivmecillinam and amoxicillin/clavulanic acid
PACUTI: Pivmecillinam with amoxicillin/clavulanic acid for step down oral therapy in febrile UTIs caused by ESBL-producing Enterobacterales
PP: Per protocol
RCT: Randomised controlled trial
SC: Trial steering committee
TMX: Trimethoprim–sulfamethoxazole
UTI: Urinary tract infections
SUSAR: Suspected unexpected serious adverse reaction

## Footnotes

1 EPE refers to ESBL-producing *Enterobacterales*. This includes *Escherichia coli*, *Klebsiella pneumoniae*, *Proteus mirabilis*, *Klebsiella oxytoca* and *Citrobacter koseri*.

2 Susceptibility for pivmecillinam in the study is based on zone diameter breakpoints for pyelonephritis (≥ 20 mm) which was received by personal communication with professor Christian Giske, the chairman of the European Committee of Antimicrobial Susceptibility Testing (EUCAST) [26].

3 EPE-active intravenous antibiotics refers to EUCAST susceptibility testing and will most often be piperacillin-tazobactam, meropenem or imipenem-cilastatin in the Swedish setting, and less often aminoglycosides or newer beta-lactamase-inhibitor-containing beta-lactam antibiotics [27]. Participants who have only received one dose of EPE-active intravenous antibiotics are also eligible and are considered within the “1–5 days” of antibiotics. Patients may only be recruited and randomised once in this trial.

4 The study treatment duration includes both the initial intravenous treatment and the allocated follow-up treatment so that the total duration adds up to 7 or 10 days. The choice between control drugs is based on the standard of care (SOC) principle, i.e. it is up to the treating physician to make the final decision. We do provide an algorithm for control drug choice in the protocol based on susceptibility testing of each clinical isolate, comorbidities, estimated glomerular filtration rate (eGFR) and guidelines by the Swedish infectious disease society [27]. See Table 1. The treating physician is however also required to consider any patient reported drug allergies and co-treatment with interacting drugs (see  Table 2) that may make one control treatment more suitable than another (or may, rarely, inhibit the allocation to any of the control drugs).

5 We advise the dosing of antibiotics to be adjusted in patients with renal insufficiency so that ciprofloxacin is given 500 mg once daily when eGFR is below 40 mL/min, TMX is given 400 mg/80 mg twice daily when eGFR is 20–40 mL/min and ertapenem is given 0.5 g once daily when eGFR is below 30 mL/min, and amoxicillin–clavulanic acid is given 500/125 mg twice daily when eGFR is below 40 mL/min.

6 The protocol standard is treatment with ertapenem as outpatient parenteral antimicrobial therapy (OPAT), but keeping patients admitted to hospital is not prohibited in the trial, and the same goes for PAC, ciprofloxacin and TMX.
